# The Therapeutic Potential of Monocyte/Macrophage Manipulation in the Treatment of Chemotherapy-Induced Painful Neuropathy

**DOI:** 10.3389/fnmol.2017.00397

**Published:** 2017-11-27

**Authors:** Karli Montague, Marzia Malcangio

**Affiliations:** Wolfson Centre for Age-Related Diseases, Guy’s Hospital Campus, King’s College London, London, United Kingdom

**Keywords:** monocyte, macrophage, chemotherapy-induced painful neuropathy (CIPN), chemokine, cytokine, therapy

## Abstract

In cancer treatments a dose-limiting side-effect of chemotherapeutic agents is the development of neuropathic pain, which is poorly managed by clinically available drugs at present. Chemotherapy-induced painful neuropathy (CIPN) is a major cause of premature cessation of treatment and so a greater understanding of the underlying mechanisms and the development of novel, more effective therapies, is greatly needed. In some cases, only a weak correlation between chemotherapy-induced pain and neuronal damage is observed both clinically and preclinically. As such, a critical role for non-neuronal cells, such as immune cells, and their communication with neurons in CIPN has recently been appreciated. In this mini-review, we will discuss preclinical evidence for the role of monocytes/macrophages in the periphery in CIPN, with a focus on that which is associated with the chemotherapeutic agents vincristine and paclitaxel. In addition we will discuss the potential mechanisms that regulate monocyte/macrophage–neuron crosstalk in this context. Informed by preclinical data, we will also consider the value of monocytes/macrophages as therapeutic targets for the treatment of CIPN clinically. Approaches that manipulate the signaling pathways discussed in this review show both promise and potential pitfalls. Nonetheless, they are emerging as innovative therapeutic targets with CX_3_CL_1_/R_1_-regulation of monocyte/macrophage–neuron communication currently emerging as a promising front-runner.

## Introduction

Chemotherapy-induced painful neuropathy (CIPN) is a dose-limiting side-effect of chemotherapeutic agents including taxanes, vinca alkaloids, and platinum-based compounds (Seretny et al., 2014). Currently available analgesics such as Gabapentin shows limited efficacy for CIPN and begets numerous undesirable side-effects themselves such as dizziness and nausea (Bloodworth, 2005; Dougherty et al., 2007). Consequently, chemotherapy is often prematurely terminated, which can jeopardize treatment success. The development of novel, more effective analgesics, which requires advances in our understanding of the underlying mechanisms of CIPN and thus identification of innovative targets, is therefore greatly needed. CIPN was initially considered to depend entirely on the responses of neurons injured by chemotherapeutic agents. Preclinical evidence, however, has uncovered a critical role of immune cell communication with neurons in the underlying mechanisms of CIPN. Indeed, a key observation indicating that neurons alone do not regulate CIPN is that pain can still arise in the absence of extensive neuronal damage, which is the case for the vinca alkaloid vincristine (Old et al., 2014). It is well-established that chemotherapeutic agents stimulate the immune system (Zitvogel et al., 2008) and so it is unsurprising that recent investigation has given consideration to immune cells in CIPN. Indeed, microglial communication with neurons in the spinal cord dorsal horn, through alterations in their expression and release of cytokines and chemokines, has been shown to be pivotal in several chronic pain states including CIPN (Malcangio, 2016; Montague and Malcangio, 2017). In some preclinical models of CIPN, however, immune cells in the central nervous system do not appear to orchestrate pain and immune cell signaling in the periphery, at the site of injury, plays a more pertinent role (Old et al., 2014).

Most chemotherapeutic agents do not penetrate the blood–brain barrier (BBB) but can cross the blood–nerve barrier (BNB) where they accumulate in dorsal root ganglia (DRG) and peripheral nerves, exerting toxicity that is exacerbated by the absence of a lymphatic system in the endoneural compartment (Weimer, 2003; Balayssac et al., 2005; Cavaletti et al., 2008). Penetration of the BNB can be attributed to relatively low levels of *P*-glycoprotein transporter activity, which limits the efficiency of toxin removal (Balayssac et al., 2005). As well as the intrusion of toxins, immune cells also infiltrate into peripheral nervous tissue. The penetration of toxins and immune cells through the BNB is exacerbated as a consequence of BNB breakdown by matrix metalloproteinases (MMPs), some of which are upregulated by chemotherapeutic agents (Peters et al., 2007). The peripheral nervous system is therefore considerably more susceptible than the central nervous system to chemotherapy-associated toxicity.

In this mini-review, we consider the role of immune cells in the periphery, specifically monocytes/macrophages, and consider the therapeutic potential of their manipulation for the prevention and/or treatment of pain in CIPN. Neuropathic pain that is associated with different chemotherapeutic agents is likely to be regulated by distinct underlying mechanisms. Here, we will focus predominantly on preclinical models of vincristine and paclitaxel neuropathic pain.

## Monocyte/Macrophages and Pain-Like Behavior In Preclinical Cipn

Monocytes are heterogenous, plastic blood cells that monitor environmental changes and alter their phenotype accordingly, differentiating into either inflammatory or anti-inflammatory subsets (Ingersoll et al., 2011; Yang et al., 2014). The primary role of monocytes was initially considered to be most prominent under steady-state conditions, with monocytes infiltrating into tissue and differentiating into tissue-resident macrophages, which serve the function of clearing cellular debris (Mueller et al., 2003). Monocyte phenotype and function, however, are now known to be more extensive, with specific subtypes possessing distinct pathophysiological functions (Yang et al., 2014). “Classic” inflammatory monocytes, for example, express a specific subset of toll-like receptors (TLRs) as well as the chemokine receptor CCR_2_, which regulates recruitment of monocytes to sites of injury/inflammation (Kurihara et al., 1997; Ginhoux and Jung, 2014). At present, monocytes are most commonly identified according to their expression of the inflammatory marker lymphocyte antigen 6 complex C (Ly6C). Ly6C-positive (+) monocytes are considered to possess an inflammatory phenotype and express high levels of CCR_2_ while “patrolling,” Ly6C-negative (-) monocytes are conventionally negative for CCR_2_ but instead express an alternative chemokine receptor – CX_3_CR_1_, which is exclusively activated by CX_3_CL_1_ (Si et al., 2010; Clark and Malcangio, 2014). At a steady state, Ly6C^+^${\mathrm{CCR}}_{2}^{+}$ monocytes differentiate into a Ly6C^-^CX_3_${\mathrm{CR}}_{1}^{+}$ phenotype in the circulation, which patrol the endothelium (Carlin et al., 2013). Under adverse conditions, however, CX_3_CR_1_ signaling in monocytes mediates their rapid infiltration through the endothelium and into tissue where they differentiate into macrophages (Auffray et al., 2007).

Infiltration of monocytes into the DRG and sciatic nerve, where they differentiate into inflammatory macrophages, has been observed in several preclinical models of CIPN and corresponds with model-associated pain. In rats treated with the taxane paclitaxel, for example, the number of macrophages in the DRG is significantly elevated concurrent with the development of cold hyperalgesia and mechanical hypersensitivity (Peters et al., 2007). The association between monocyte/macrophage infiltration and some preclinical models of CIPN has been reinforced pharmacologically. Minocycline, for example, which alongside other actions, inhibits monocyte/macrophage infiltration (Liu et al., 2010), has been shown to alleviate oxaliplatin-induced pain (Boyette-Davis and Dougherty, 2011). Furthermore, depletion of macrophages using liposome-encapsulated clodronate (LCL) reduces paclitaxel-associated mechanical hypersensitivity. LCL concurrently lowers the paclitaxel-induced increase in macrophages in the DRG as well as the expression of the proinflammatory cytokine tumor necrosis factor alpha (TNFα), suggesting that macrophages comprise a feedback mechanism that increases monocyte/macrophage infiltration and proinflammatory cytokine expression (Zhang et al., 2016). This association between monocyte/macrophage infiltration and pain also applies to preclinical CIPN induced by vinca alkaloids. In a vincristine model of CIPN for instance, mechanical hypersensitivity and the elevation of macrophages in the DRG and sciatic nerve occur concomitantly within 24 h of the first vincristine dose and remain elevated during, and a few weeks after, treatment completion (Old et al., 2014). Furthermore, when mechanical hypersensitivity is no longer present a few weeks after treatment cessation, the number of macrophages in the DRG and sciatic nerve is also no longer elevated, suggesting that macrophage elevation in the DRG and sciatic nerve is functionally linked to pain-like behavior. Indeed, transient depletion of macrophages using LCL significantly delays the onset of vincristine-induced mechanical hypersensitivity (Old et al., 2014).

The strong association between increased monocyte/macrophage infiltration into the DRG and sciatic nerve with pain-like behavior in several preclinical models of CIPN suggests that manipulating monocytes/macrophages in the periphery has prophylactic and therapeutic potential for CIPN associated with some chemotherapeutic agents and could form the basis of innovative therapies. In order to identify the most efficacious approach, an understanding of how monocytes/macrophages communicate with neurons in response to chemotherapy treatment is essential. An established means by which macrophages communicate with neurons is chemokine signaling. Indeed, evidence for the role of chemokine-mediated macrophage–neuron communication as well as monocyte–endothelium crosstalk in some preclinical models of CIPN has strengthened considerably in the last few years.

## Chemokine-Mediated Monocyte/Macrophage–Neuron Communication

### CX_3_CL_1_/R_1_ Signaling in CIPN

At present, the chemokine that perhaps appears to have the most authentic role in mediating monocyte/macrophage–neuron crosstalk in the periphery in some models of CIPN is CX_3_CL_1_. CX_3_CL_1_ (fractalkine) is the only member of the CX_3_C family of chemokines that was first described 20 years ago (Bazan et al., 1997). CX_3_CL_1_ exists as both membrane-tethered and soluble forms and in the periphery is expressed by endothelial cells (Imaizumi et al., 2004). Soluble, peripheral CX_3_CL_1_ is generated constitutively by cleavage mediated by the endothelial-derived metalloprotease ADAM10, while ADAM17 regulates cleavage during adverse conditions (Hundhausen et al., 2003; Hurst et al., 2009). Unlike other chemokines, for which signaling is promiscuous (Bennett et al., 2011), CX_3_CL_1_ exclusively activates CX_3_CR_1_, which is expressed by patrolling monocytes (Jung et al., 2000). Endothelial CX_3_CL_1_ activation of CX_3_CR_1_ in monocytes plays a role in, although is not essential for, monocyte crawling along the endothelium (Carlin et al., 2013), while soluble CX_3_CL_1_ activation of CX_3_CR_1_ promotes their transendothelial migration (Schwarz et al., 2010).

**FIGURE 1:**
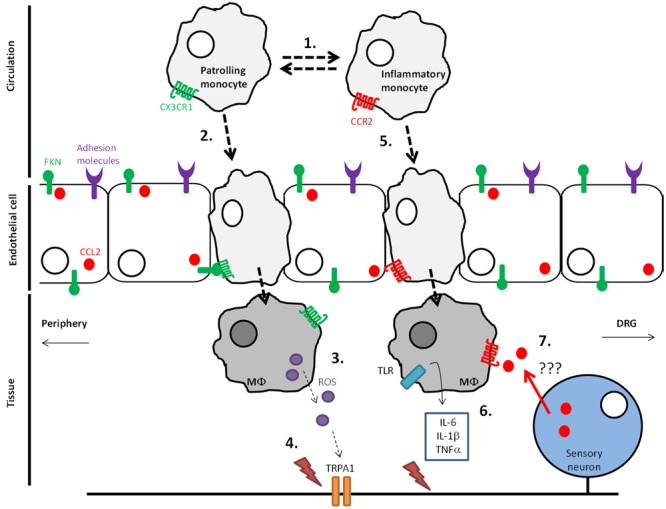
Monocyte infiltration into DRG and peripheral nervous tissue in vincristine-induced neuropathic pain. (1) Under steady-state conditions Ly6C^-^CX_3_${\mathrm{CR}}_{1}^{+}$${\mathrm{CCR}}_{2}^{–}$ monocytes, which patrol the endothelium, dominate over inflammatory Ly6C^+^CX_3_${\mathrm{CR}}_{1}^{–}$${\mathrm{CCR}}_{2}^{+}$ monocytes in the circulation. (2) During adverse conditions such as those that occur following chemotherapy exposure, CX_3_${\mathrm{CR}}_{1}^{+}$ monocytes infiltrate through the endothelium and into nervous tissue in a soluble CX_3_CL_1_-dependent manner, which is produced by cleavage mediated by ADAM17 in adverse conditions (3). (4) CX_3_${\mathrm{CR}}_{1}^{+}$ monocytes differentiate into macrophages in nervous tissue where they express reactive oxygen species (ROS). ROS activation of TRPA1 channels in peripheral nerve fibers evokes pain. (5) During adverse conditions, Ly6C^+^${\mathrm{CCR}}_{2}^{+}$ monocytes also infiltrate into peripheral nervous tissue in a CCL_2_-dependent manner. (6) ${\mathrm{CCR}}_{2}^{+}$ macrophages in tissue generate cytokines, which activate nociceptive fibers. (7) Indirect evidence suggests the presence of CCL_2_ in the periphery can serve as a positive feedback mechanism by which infiltration of ${\mathrm{CCR}}_{2}^{+}$ monocytes is increased further.

CX_3_CR_1_-expressing monocytes appear to orchestrate the development of pain in a preclinical vincristine model of CIPN. Specifically, in CX_3_CR_1_ knock-out (KO) mice, there is a significant delay in the induction of mechanical hypersensitivity by vincristine that resembles the delay observed when macrophages are transiently depleted with LCL (Old et al., 2014). Concurrent with delayed mechanical hypersensitivity, a delay in monocyte infiltration into the sciatic nerve is also observed in CX_3_CR_1_ KO mice. Whereas the number of cells expressing the macrophage marker F4/80 is elevated within 1 day of the first vincristine dose in control mice, CX_3_CR_1_ KO mice do not display a significant increase in F4/80+ cells in the sciatic nerve until day 5 – the same time point at which mechanical hypersensitivity appears in these mice. Intriguingly, injury-associated monocyte/macrophage infiltration into the sciatic nerve does not appear to be affected in CX_3_CR_1_ KO mice following partial sciatic nerve ligation (Staniland et al., 2010), suggesting that the involvement of CX_3_CR_1_ signaling in monocytes/macrophages is model-specific. Although vincristine does not appear to increase endothelial expression of CX_3_CL_1_, it does increase endothelial expression of adhesion molecules, which could promote recruitment of CX_3_CR_1_-expressing monocytes and subsequent infiltration into the sciatic nerve. Here, macrophages generate reactive oxygen species (ROS) in response to vincristine in a CX_3_CR_1_-dependent manner, which in turn activate TRPA_1_ channels thus evoking pain (**Figure 1**) (Old et al., 2014).

Manipulating CX_3_CL_1_/R_1_ signaling in monocytes/ macrophages in the sciatic nerve could thus provide a prophylactic treatment for the development of pain in CIPN. A caveat is that CX_3_CR_1_ KO is global and may not represent targeted pharmacological inhibition. To strengthen the prophylactic potential of CX_3_CL_1_/R_1_ clinically, pharmacological studies must support transgenic studies. Indeed, pharmacological inhibition of CX_3_CL_1_/R_1_ signaling in monocytes/macrophages shows promise in the paclitaxel preclinical model of CIPN, with intrathecal pre-treatment of rats with a CX_3_CR_1_ neutralizing antibody significantly reducing paclitaxel-associated mechanical hypersensitivity (Huang et al., 2014). Furthermore, paclitaxel-associated monocyte infiltration into the DRG is also reduced by prophylactic administration of the antibody as is macrophage activation, as demonstrated by decreased p38 phosphorylation. The study also reports reduction in neuronal apoptosis demonstrated by reduced caspase 3 expression in the presence of the antibody (Huang et al., 2014).

Pharmacological inhibition of CX_3_CR_1_ signaling in monocytes/macrophages therefore appears to constitute a potential prophylactic treatment for CIPN associated with vincristine and paclitaxel treatment. A valuable feature of CX_3_CL_1_/R_1_ signaling is its high fidelity, which limits the likelihood of unexpected side-effects. It is important to appreciate, however, that, unlike other chemokines, CX_3_CL_1_ is constitutively expressed and thus targeting CX_3_CL_1_/R_1_ signaling could disrupt critical homeostatic processes. Moreover, the eventual development of pain in CX_3_CR_1_ KO mice suggests that the role of CX_3_CL_1_ signaling in monocytes/macrophages changes over time and mechanisms underlying vincristine-induced neuropathic pain, for example, are dynamic. Targeting CX_3_CR_1_ signaling in monocytes/macrophages could therefore provide a prophylactic treatment, which could form part of a tailored, combination therapy. Alternative mediators of monocyte/macrophage communication with neurons should therefore be identified in order to uncover additional potential targets for treating vincristine pain in patients at different stages of chemotherapy and indeed CIPN associated with other chemotherapeutic agents. Indeed, monocytes/macrophages in the periphery express other chemokines and their receptors, which have been implicated in several preclinical models of chronic pain.

### Alternative Macrophage-Derived Chemokines and Chronic Pain

Chemokine (C-C motif) ligand 4 (CCL_4_), otherwise known as macrophage inflammatory protein 1b is an alternative chemokine that is expressed by macrophages (Chong et al., 2002). CCL_4_ signals via the CCR_5_ receptor that is also expressed by macrophages, however, unlike CX_3_CL_1_/R_1_ signaling, the CCL_4_/R_5_ partnership does not display fidelity (Jones et al., 2011). CCL_4_ activation of CCR_5_ in macrophages has been associated with chronic pain induced by surgical damage. For instance, following partial sciatic nerve ligation, CCL_4_ mRNA is significantly elevated in macrophages alongside pain-like behavior, and the inhibition of CCL_4_ using local application of a neutralizing antibody in the sciatic nerve alleviates surgical-induced pain (Saika et al., 2012). CCL_4_/R_5_ signaling in peripheral macrophages has not been specifically investigated in the context of preclinical CIPN, however, recent evidence has indicated that the expression of CCL_4_ increases centrally following paclitaxel treatment, yet intriguingly, decreases in the DRG (Makker et al., 2017) making it only a weak candidate for a peripheral mediator of paclitaxel-induced painful neuropathy.

Monocytes under inflammatory conditions express CCR_2_ (Yang et al., 2014). Indeed, existing evidence implicates neuronal CCL_2_/R_2_ signaling in several chronic pain models, including CIPN. For instance, neuronal CCL_2_/R_2_ signaling in the DRG has been strongly implicated in chemotherapy pain and CCL_2_/R_2_ signaling is known to mediate neuron–macrophage communication (Kwon et al., 2015). In a preclinical paclitaxel pain model for instance, expression of both CCL_2_ and CCR_2_ increases in DRG neurons alongside the development of mechanical hypersensitivity (Zhang et al., 2013). The increase in macrophages in the DRG in this model is well-established and it is therefore plausible that elevated CCL_2_ could also activate CCR_2_ expressed by macrophages in addition to DRG neurons (White et al., 2005). What remains to be validated, however, is whether or not neurons release the CCL_2_ that they have been shown to express. Nonetheless, increased CCL_2_ in the DRG could constitute a feed-forward mechanism by which CCL_2_ stimulates further monocyte/macrophage infiltration (Groh et al., 2010). Indeed, intrathecal administration of an anti-CCL_2_ antibody not only blocks paclitaxel-associated pain behavior, but also reduces the associated monocyte/macrophage infiltration into the DRG, although whether this effect is direct or indirect, as well as the precise site of action, has yet to be established (Zhang et al., 2016).

Currently, tangible evidence for a role of CCL_2_/R_2_ signaling in monocytes/macrophages in the periphery in some preclinical models of CIPN has not been obtained. However, the involvement of CCL_2_/R_2_ signaling in chronic pain and the expression of CCR_2_ in inflammatory macrophages make CCL_2_/R_2_ signaling in macrophages an intuitive candidate for the regulation of CIPN, particularly at later stages when the role of CX_3_CL_1_/R_1_-mediated monocyte/macrophage signaling appears to be less pertinent.

## Cytokine Production By Macrophages In Peripheral Tissue

In addition to chemokines, macrophages in the periphery express and release proinflammatory cytokines, which have well-established pronociceptive effects (Sommer and Kress, 2004). Cytokines are diverse glycoproteins that are predominantly secreted by immune cells such as macrophages. Interleukin-6 (IL-6), IL-1β, and TNFα are the most consistently elevated cytokines in response to damage and inflammation.

### Interleukin-6 (IL-6)

The proinflammatory cytokine IL-6 is secreted predominantly by macrophages in adverse conditions. IL-6 signals classically via membrane-bound IL-6R, which is expressed by neurons (Erta et al., 2012). IL-6R can also exist as a soluble form following cleavage by either ADAM10 or ADAM17 and signaling via the soluble receptor, which is referred to as IL-6 *trans*-signaling, is associated with monocytes of a proinflammatory ${\mathrm{CCR}}_{2}^{+}$ phenotype (Scheller et al., 2011).

The expression of IL-6 by macrophages in peripheral tissue has been associated with vincristine-induced pain behavior preclinically. Following one cycle of vincristine treatment, the development of pain behavior in mice is accompanied by an elevation of macrophages in the DRG and sciatic nerve, which also display positive immunoreactivity for IL-6 (Kiguchi et al., 2008b). Furthermore, inhibition of IL-6 by local injection of a neutralizing antibody in the vicinity of the sciatic nerve results in a significant alleviation of mechanical hypersensitivity (Kiguchi et al., 2008b). IL-6, however, has also been suggested to potentially possess anti-inflammatory properties (Scheller et al., 2011) and so the side-effect profile associated with its inhibition could be problematic. Nonetheless, its production by macrophages and subsequent activation of neurons could provide an additional mechanism for monocyte/macrophage–neuron communication in vincristine-induced neuropathic pain.

### Interleukin 1β (IL-1β)

Interleukin 1β is a proinflammatory cytokine, which signals via the IL-1 receptor 1. As is the case with IL-6, IL-1β signaling also has the capacity to trigger macrophage differentiation (Schenk et al., 2014). Due to its ability to rapidly excite nociceptive fibers, IL-1β was one of the first cytokines to be associated with chronic peripheral pain conditions, with IL-1β KO mice demonstrating resistance to surgery-induced pain (Kleibeuker et al., 2008). IL-1β is expressed by bone marrow-derived macrophages in response to a variety of chemotherapeutic agents. Agents such as vincristine, cisplatin, paclitaxel, melphalan, and methotrexate, for example, have all been shown to stimulate IL-1β production in LPS-primed bone marrow-derived macrophages (Wong et al., 2014). The contribution of such production to chemotherapy-induced pain, however, as yet to be determined.

### Tumor Necrosis Factor Alpha (TNFα)

Tumor necrosis factor alpha is also produced and secreted by a number of cell types, however, in the context of chronic pain, elevation of TNFα occurs predominantly in macrophages (Sommer and Schäfers, 1998). As is the case with ILs, TNFα also rapidly and directly stimulates and sensitizes A- and C-fibers (Schäfers and Sorkin, 2008), providing a potential pathway by which monocyte/macrophage–neuron communication could occur. Alterations in TNFα expression both peripherally and centrally have been observed in certain models of preclinical CIPN. Specifically, increases in expression of TNFα in the sciatic nerve and spinal cord have been found to occur alongside pain-like behavior in vincristine rat and mouse models, respectively (Kiguchi et al., 2008a; Muthuraman et al., 2011). Not only are macrophages a major source of TNFα, but they are also responsive to it, with TNFα stimulation of macrophages resulting in increased cytokine production (Parameswaran and Patial, 2010). TNFα signaling in macrophages could therefore constitute a feed-forward mechanism, which maintains cytokine production and chronic communication with neurons.

## Toll-Like Receptor Activation of Macrophages

As well as considering monocyte/macrophage signaling, one must also consider their activation when identifying approaches for the manipulation of monocytes/macrophages. One of the mechanisms by which the release of inflammatory mediators from macrophages is triggered is via the activation of TLRs expressed at their cell surface. Macrophages express an array of TLRs, which can stimulate cytokine release. Activation of TLR4, for example, results in the release of both TNFα and IL-1β, while stimulation of TLRs 3, 9, and 7 stimulate the release of IL-1α and 1β (Nicotra et al., 2012). TLR activation of macrophages has been associated with the regulation of chronic pain as it constitutes a feed-forward mechanism, with activation of macrophages via TLRs 3, 7, and 9 signaling resulting in an consequential upregulation of TRPV_1_ expression in DRG neurons (Diogenes et al., 2011).

TLR4-mediated activation of macrophages in the DRG has been shown to be involved in preclinical paclitaxel-associated painful neuropathy. In this model, in which macrophages are elevated in the DRG alongside the occurrence of mechanical hypersensitivity, administration of a TLR4 antagonist, LPS-RS, alongside paclitaxel, significantly reduces paclitaxel-associated pain behavior as well as monocyte/macrophage infiltration into the DRG (Zhang et al., 2016).

Targeting TLR signaling, however, is likely to be complicated. Not only do macrophages express numerous TLRs, but neurons in the DRG also express TLRs 1, 2, 3, 4, 5, 6, and 9 (Ochoa-Cortes et al., 2010). The analgesic effects of targeting TLRs could therefore be equally attributed to inhibition of neuronal TLR activation.

## Conclusion: the Therapeutic Potential of Monocyte/Macrophage Manipulation

Currently available analgesics show a limited efficacy at treating CIPN. The underlying mechanisms are poorly understood and are likely to vary with different chemotherapeutic agents. We are beginning to uncover and understand the importance of monocyte/macrophage–neuron communication in the mediation CIPN and accumulating preclinical evidence is indicative of its promising potential as an innovative prophylactic and therapeutic strategy.

Although monocyte/macrophage manipulation for the treatment of CIPN remains preclinical to date, the approach has entered clinical trials in the context of other pathological conditions. A monoclonal antibody against colony stimulating factor 1 (CSF-1), also known as macrophage stimulating factor, which regulates the differentiation of macrophages, has been used in clinical trials for treating solid tumors and appears to be well-tolerated (Panni et al., 2013). Most notably, a humanized monoclonal antibody against CX_3_CL_1_ has also been found to be safe and well-tolerated in the clinic when used in Rheumatoid Arthritis and Crohn’s Disease patients (Imai and Yasuda, 2016). The application of monocyte/macrophage manipulation to CIPN patients is therefore feasible in light of patients’ tolerability to such an approach in other contexts.

Novel pain therapies should not themselves jeopardize the success of chemotherapy and should have minimal side-effects in order to avoid reducing the patient’s quality of life further. CX_3_CL_1_/R_1_ displays a high fidelity signaling relationship, which is likely to limit the occurrence of unexpected side-effects. Furthermore, the role of CX_3_CL_1_/R_1_-mediated monocyte/macrophage–neuron communication in the periphery in vincristine- and paclitaxel-associated painful neuropathy specifically is arguably supported by the most tangible evidence at present and the humanized monoclonal antibody against CX_3_CL_1_ is well-tolerated clinically. The manipulation of monocytes/macrophages via manipulation of CX_3_CL_1_/R_1_ signaling therefore appears to be the current front-runner for prophylactic treatment of neuropathic pain in patients treated with vincristine and paclitaxel. Whether this also applies to other chemotherapeutic agents, however, remains unknown. The next step of this exciting journey is to identify other approaches for monocyte/macrophage manipulation that will compliment CX_3_CL_1_/R_1_ inhibition, allowing us to develop tailored therapies that can be used to treat patients at various stages of chemotherapy treatment.

## Author Contributions

All authors listed have made a substantial, direct and intellectual contribution to the work, and approved it for publication.

## Conflict of Interest Statement

The authors declare that the research was conducted in the absence of any commercial or financial relationships that could be construed as a potential conflict of interest.
